# Core competencies in genetics for healthcare professionals: results from a literature review and a Delphi method

**DOI:** 10.1186/s12909-019-1456-7

**Published:** 2019-01-11

**Authors:** Alessia Tognetto, Maria Benedetta Michelazzo, Walter Ricciardi, Antonio Federici, Stefania Boccia

**Affiliations:** 1 Università Cattolica del Sacro Cuore, Sezione di Igiene, Istituto di Sanità Pubblica, Roma, Italy; 2 Istituto Superiore di Sanità, Roma, Italy; 3 Direzione Generale Prevenzione Sanitaria, Ministero della Salute, Roma, Italy; 4 Fondazione Policlinico Universitario A. Gemelli IRCCS, UOC Igiene Ospedaliera, Roma, Italy

**Keywords:** Public health genomics, Delphi survey, Genetics, Health education, Curriculum, Medical education, Genetics competencies for health care professionals

## Abstract

**Background:**

Advances in genetics and genomics require that healthcare professionals manage and incorporate new technologies into the appropriate clinical practice. The aim of this study was to identify core competencies in genetics for non-geneticists, both physicians and non-physicians.

**Methods:**

We performed a literature review by searching MEDLINE, SCOPUS, and ISI Web of Science databases to identify studies reporting competencies in genetics in terms of knowledge, attitudes and abilities for non-genetic healthcare professionals. Furthermore, we conducted a survey according to a modified Delphi method, involving genetics experts to evaluate the competencies to be included as items of the curricula.

**Results:**

Three eligible documents were identified and 3 Delphi rounds were carried out to reach a consensus on the competencies to be incorporated in the curricula. With reference to the curriculum for physicians, 19 items were included in the knowledge domain, 3 in the attitudes and 10 in the abilities domain. We developed two different curricula for non-physicians: one specific for those working in genetic services (20 items in the knowledge domain, 3 in the attitudes and 12 in the abilities) and one for those not working in genetic services (10 items in the knowledge domain, 3 in the attitudes and 2 in the abilities).

**Conclusions:**

We developed 3 curricula in genetics addressed to non-genetic healthcare professionals. They differ in the “knowledge” and “abilities”, while the “attitudes” are the same for all the healthcare professionals. Although some concerns about the generalizability of the findings could arise due to the Italian perspective, we envisage the curricula can be used for genetics educational programs in several contexts.

## Background

Over the last decades, new DNA sequencing technologies have been offered at increasingly reduced costs [1, 2]. This has led to a rapid spread of genetic tests utilization in the clinical practice [3]. The spread of this type of testing runs in parallel with an increasing inappropriateness that might be partly related to a lack of competencies of healthcare professionals that refer patients to genetic services [4–6]. Non-genetic healthcare providers themselves feel inadequately qualified to decide whether genetic testing is appropriate, to perform a genetic risk assessment and to interpret the genetic test results [7]. Khoury et al. highlighted to what extent the translation of genomics knowledge into clinical practice is a challenging phase of translational research [8]. Thus, the potential benefits of a genetic test can be affected by the lack of a proper education and training of non-genetic healthcare professionals [9].

In the Italian framework, different authors have reported the need to improve the genomic literacy of non-genetic healthcare workers [10–12] differentiating between physicians and non-physicians, since their careers are widely different in terms of educational programmes [13].

The predefinition of a set of core competencies is essential for the development of an educational programme [14]. A “competence” is made up of different aspects, including knowledge, relational attitudes and practical abilities [15, 16] and it is more complex than each single aspect as a standalone. In the educational context, the modifier “core” refers to that set of competencies that are identified as essential [17]. The definition of the core competencies for a certain professional category should be based on the assessment of educational needs, achievable through an appropriate reference to the existing evidence and through the involvement of an adequate group of experts [18, 19].

Our study was based on the hypothesis that a specific healthcare professional education is the first step for the appropriate implementation of genetics/genomics in clinical practice to guide decisions on prevention, diagnosis and treatment of patients. In this conceptual framework, our research aimed to identify the core competencies in genetics, developed in a curriculum-style structure, for both non-genetic physicians and non-physicians. As to the latter, we differentiated between healthcare professionals who work in genetic services and the others. An Italian perspective was adopted to develop these curricula. We targeted at those professionals operating in our healthcare system organization and excluded categories, like non-physician genetic counsellors, who do not operate in the Italian health system.

In order to achieve this goal, we performed a literature review to identify the potential competencies that may contribute to the core set. Afterwards, we conducted a survey according to a modified Delphi method, involving a group of genetics experts to evaluate the competencies to be included as items of the curricula.

## Methods

### Literature review

#### Search strategy and study selection

We searched the MEDLINE, SCOPUS and ISI Web of Science databases, including the following search terms: “genetics”, “genomics”, “health professional”, “healthcare professional”, “clinician”, “doctor”, “professional education”, “competence”, “continuing education”, “genetics curriculum”.

The search was limited to English or Italian written articles published from January 1st, 2007 to January 1st, 2018. We selected the year 2007 as the starting point of the search period because the Italian Task Force on Public Health Genomics was launched at the end of 2016, thereby underlying the importance of genetics and genomics education in the Italian context.

We performed an extensive cross-check of the references from the original studies using a snowball approach to find additional studies. Two investigators (AT and MBM) screened the records (titles and abstracts) that were taken into account for a full-text analysis in case they fulfilled the inclusion criteria. Any disagreements were resolved through discussion and review by a supervisor researcher (SB). The review was drafted in accordance with the PRISMA guidelines [20].

#### Inclusion and exclusion criteria

The inclusion criteria required that the articles reported: 1) set of competencies in genetics for graduated healthcare professionals; 2) set of competencies according to the three domains of theoretical knowledge, relational attitudes and practical abilities; 3) description of the methodology used to identify the competencies. Articles that reported curricula for categories of healthcare professionals that are not currently present in Italy, such as non-physician genetic counsellors, were excluded.

#### Data extraction

Qualitative data extraction was performed by two investigators (AT and MBM) who collected the following information from each article: name of the scientific society, publication year, country (if any) to which the article was referred, target professionals. We collected the competencies reported in the retrieved studies according to the three domains of theoretical knowledge, relational attitudes and practical abilities. We did not perform the quality assessment of the selected studies, since inclusion criteria were strict enough to avoid inclusion of low-quality reports.

### Delphi process

We used a Delphi method to process the results from the literature review and select the competencies to include in the curricula. This method was originally developed in the 1950s by the Rand Corporation and mainly implemented by Dalkey and Helmer in 1963. It is a social science technique used in qualitative research to consult experts on a specific topic thanks to serial rounds of questionnaires. After the elaboration of data resulting in each round, a new set of questions is generated, and a new group report is created. This process is performed until a consensus is reached [21].

This approach has frequently been adopted in healthcare research and education to identify the competencies that professionals should acquire on the basis of experts’ judgement [19, 22, 23].

We adopted a modified Delphi process: we provided a base of competencies retrieved from the literature instead of asking the experts to propose them. In the first round, however, experts could propose new competencies. Thirty-seven healthcare professional experts of the Italian Network for Public Health Genomics (GENISAP) were involved in the Delphi process [24]. The GENISAP is a network created to connect Italian professionals with great knowledge and experience in the field of genetics and genomics. Invitations were sent via e-mail, with the description of the study and the assurance of anonymity of their participation in the study. Disclosure of conflicts of interest was requested to the participants. In order to increase the response rate, between the rounds a reminder was sent to the participants by e-mail.

The questionnaire was divided into three sections. The first section reported demographic information: age, gender, professional qualification, years of work experience in genetics, teaching experience in genetics. In the second section, participants were asked to approve or disapprove of the organization of the competencies into the three domains of knowledge, attitudes and abilities. The third section included all the specific items that had to be evaluated by the participants. Each item could be voted in the survey to be included in the curriculum for physicians, in the curriculum for non-physicians and/or in the curriculum dedicated to non-physicians working in genetic services.

In the first round the participants were asked to assess each item as “important”, “should be modified”, or “not important”. In an additional dedicated section, modifications or integrations of the items could be proposed. If at least 70% of the participants rated the item as “important”, the item would automatically be included in the curriculum. In the same way, if at least 70% of participants considered an item “not important”, it was automatically excluded. In the remaining cases, items were proposed again to be voted in the second round, along with the modifications proposed by the participants. Two authors (AT and MBM) evaluated all modifications suggested and integrated them into the items. In the second round, the original and the modified items were reported aligned for comparison and participants could vote “yes” or “no” for the inclusion of the modified items in the curricula. As in the first round, the threshold for inclusion was 70%.

Moreover, in the first round, participants could propose additional items, which were voted in the second round with the same system as the first round. In case of non-consensus, items were modified as suggested by the participants and voted again in the third round. In the third round the participants could vote “yes” or “no” for the inclusion of the modified items into the curricula, with a 70% consensus threshold. Since a consensus was reached among respondents, the third round was the last one. At the end of the survey, results were returned to the participants.

**Fig. 1:**
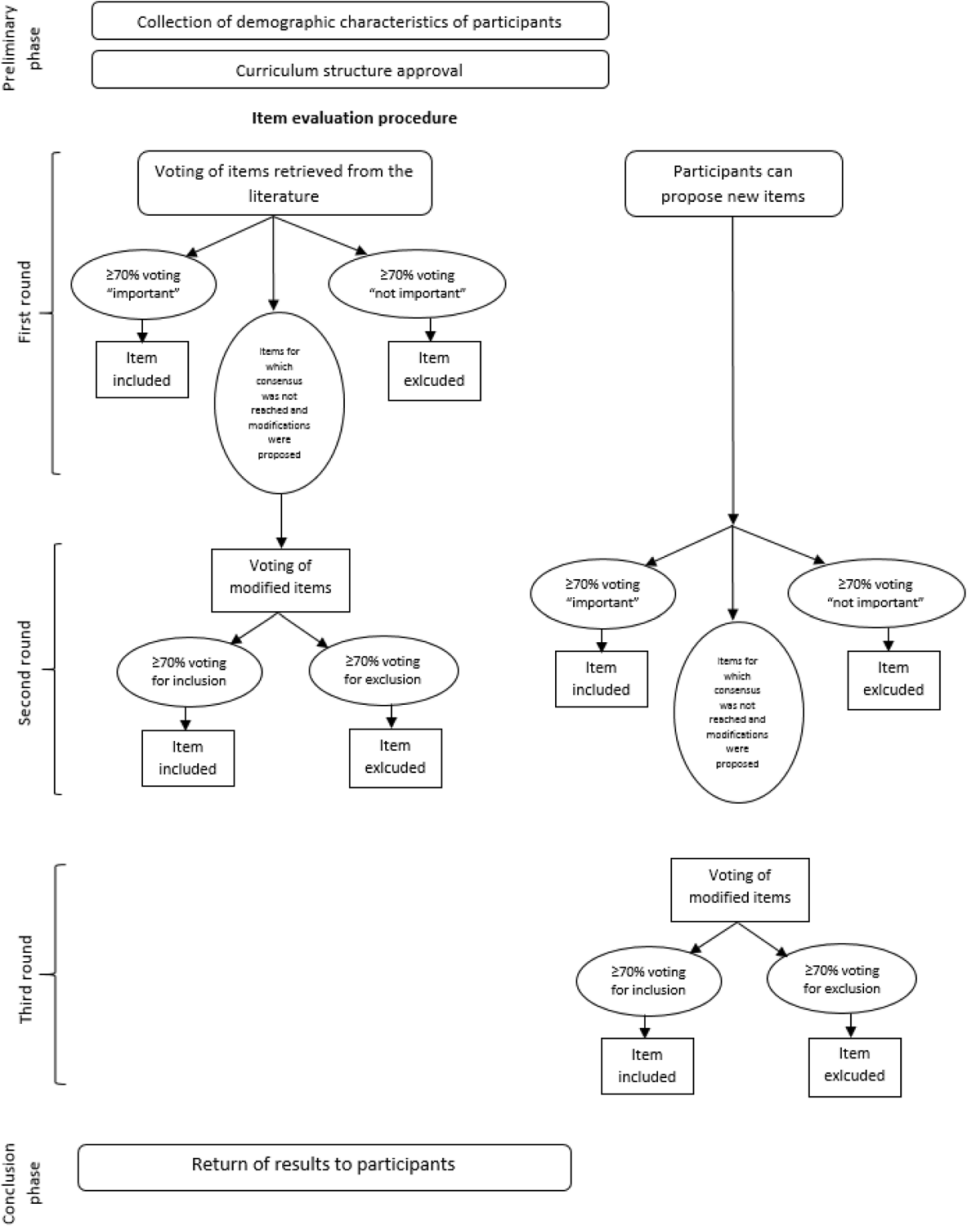
Flowchart of the steps of the Delphi process

Fig. 1 reports the Delphi process steps along with a graphic illustration of the consensus definition.

## Results

### Literature review

**Fig. 2:**
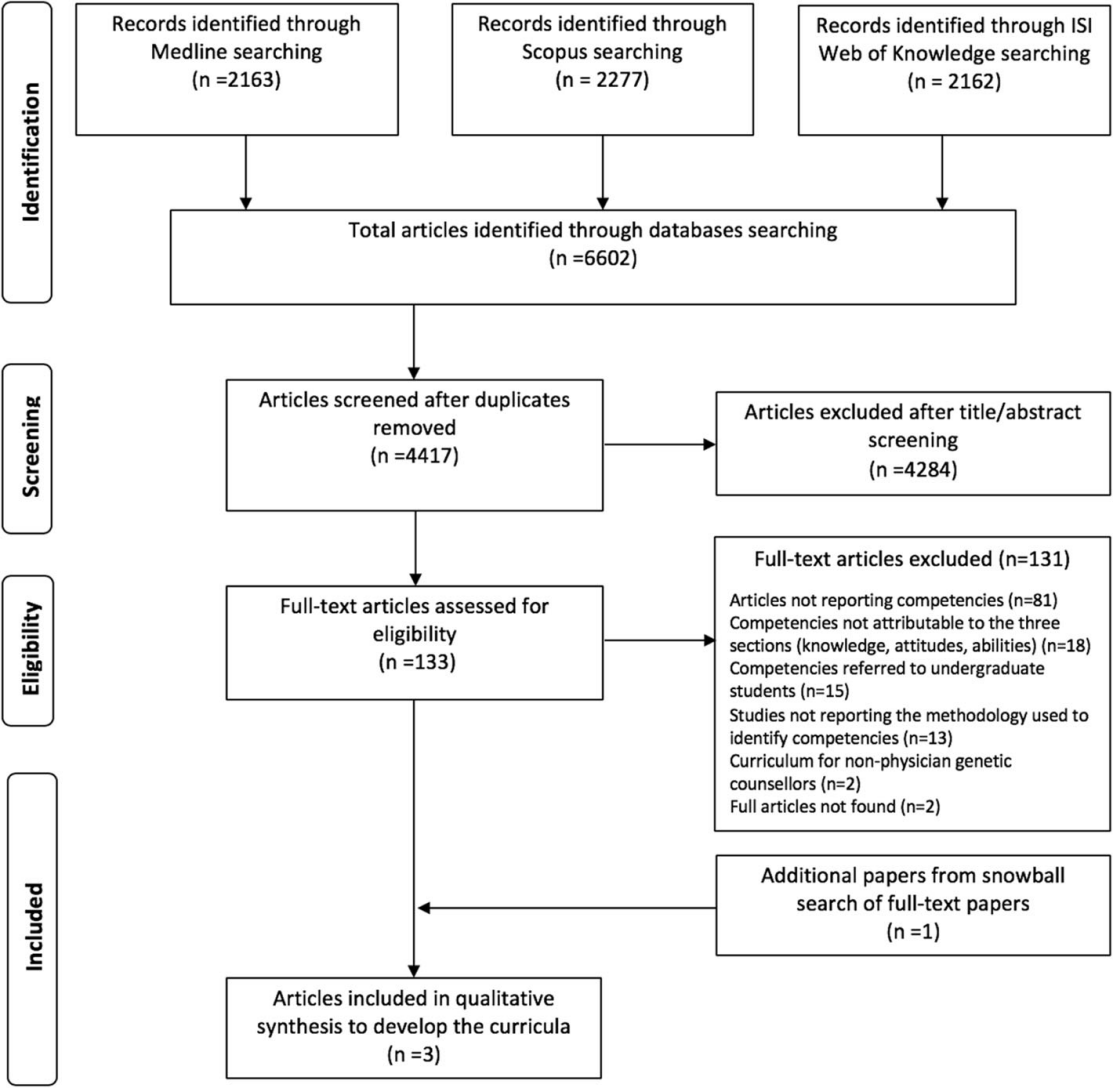
Flowchart of the bibliographic strategy and results

After removing duplicates, we identified a total of 4417 articles of which 4284 were excluded after title and abstract screening because not related to the research topic. The remaining 133 articles were assessed for eligibility and 131 were excluded because they did not meet the inclusion criteria. Figure 2 reports the flowchart of the bibliographic search strategy and the results.

Table 1 describes the main characteristics of the two studies selected through the literature review [17, 25] and of the document retrieved as a result of the snowball search [26]. These were the “*Core Competencies in Genetics for Health Professionals in Europe*” published by the European Society of Human Genetics (ESHG) [17], the “*Core Competencies in Genetics for Health Professionals”* published by the National Coalition for Health Professional Education in Genetics (NCHPEG) [25], and the “*Learning outcomes in genetics and genomics for specialty trainees in non-genetic specialties*” published by the UK National Health Service National Genetics and Genomics Education Centre (UK NHS NGGEC) [26].

**Table 1:** Characteristic of the documents retrieved through the literature review

Scientific society that produced the document	Year of publication	Country to which the document referred	Target Professionals
ESHG – Eurogentest [17]	2007	Europe	Physicians not specialized in genetics Non-physician healthcare professionals
NCHPEG [25]	2007	USA	All healthcare professionals
NHS NGGEC [26]	2015	UK	Physicians not specialized in genetics

The individual competencies from each of the three studies were unified into a single report and divided into the aforementioned three domains. The total number of the competencies was 33, of which 19 items for “knowledge”, 3 for “attitudes” and 11 for “abilities”. All these items were proposed for evaluation in the Delphi survey.

### Delphi process

**Fig. 3:**
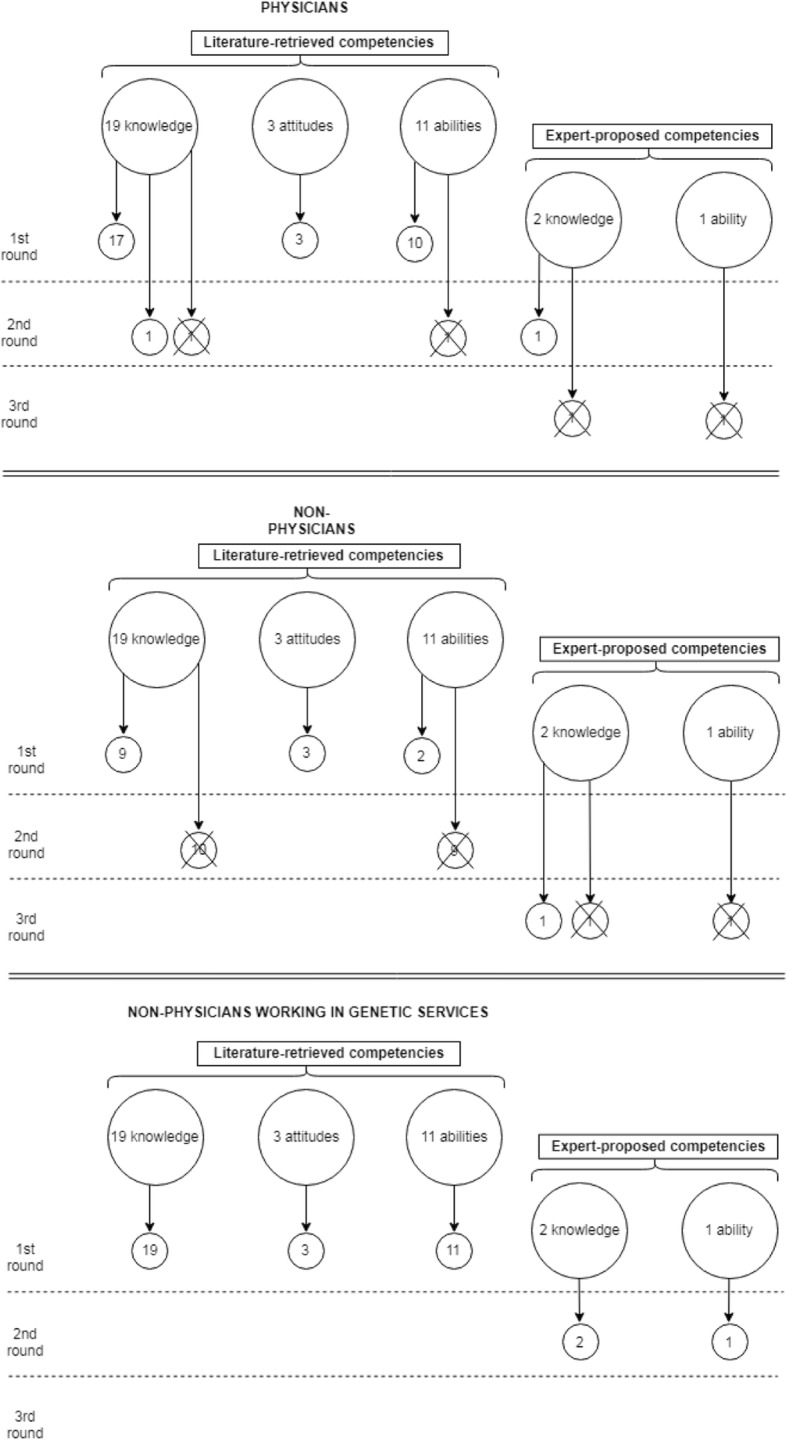
Flowchart of the results of the Delphi process

A total of 23 GENISAP members (62.2% out of 37 invited) participated in the first round, 21 (56.8%) in the second round and 12 (32.4%) in the third one. The participants of the first round, with a median age of 57 years (range 28–67), were 65.2% females and included 34.8% of geneticists, 34.8% of biologists, 17.4% of non-geneticist physicians and 13% of other healthcare professional categories. Most of the participants (78.3%) had a previous teaching experience in genetics. No disclosed conflicts of interest were reported by the experts. Figure 3 describes the results of the Delphi process.

The division into three domains (knowledge, attitudes and abilities) of the curricula structure was approved by 95.7% of the participants.

As to the curriculum for non-geneticist physicians, 30 out of the 33 items proposed were included after the first round. For the remaining 3, some modifications were proposed to be voted in the second round, after which only one was included. During the first round the participants proposed 3 new items, of which only one was included after the second round. Lastly, a total of 32 items were included in the curriculum for non-geneticist physicians: 19 concerning knowledge, 3 concerning attitudes and 10 concerning abilities (Table 2).

**Table 2:** Core curriculum for non-geneticist physicians, identified through the Delphi process

Knowledge	
1. Knowledge of the structure and function of nuclear DNA, genes and chromosomes, their organization into the genome, their replication and transmission through mitosis and meiosis	
2. Knowledge of the structure and regulation of protein-coding genes, their transcription and translation, RNA construction, protein synthesis	
3. Knowledge of the structure, function and transmission of mitochondrial DNA	
4. Knowledge of the process of DNA mutations (de novo, hereditary); knowledge of the role of these mutations as physiological or pathological events (cancer, multifactorial diseases, monogenic diseases)	
5. Understanding the difference between clinical diagnosis of disease and genetic predisposition to disease. Knowledge of the different types of genetic tests (diagnostic, predictive, test for carriers)	
6. Knowledge of transmission of hereditary diseases (autosomal dominant/recessive, X-linked, mitochondrial, chromosomal, multifactorial)	
7. Knowledge of genotype-phenotype correlations; understanding of how gene variations can influence disease presentation, its severity, and clinical manifestation (anticipation, incomplete penetrance, variable expressivity)	
8. Knowledge of the most frequent genetic variants in your professional specialty; knowledge of the clinical features and therapeutic response associated with the different variants	
9. Basic knowledge of the research approaches used to study genomic variants and their correlation with clinical data	
10. Understanding of the importance of the three-generation family history in assessing predisposition to disease	
11. Knowledge of the role of genetic factors in disease prevention	
12. Understanding of the role of behavioural, social, and environmental factors that modify or influence genetics in the manifestation of complex diseases	
13. Knowledge of the organization of genetic services	
14. Knowledge of the potential physical and/or psychosocial benefits and risks of genetic information for individuals in the context of the family and community, here included also the possibility of preventive measures such as reproductive options for mutation carriers	
15. Knowledge of the genetic approaches to treatment (including pharmacogenomics and gene therapy)	
16. Knowledge of the indications for genetic testing and referral to genetic specialists	
17. Knowledge of the principal methodologies for genetic sampling, laboratory techniques with their pros and cons, and knowledge of the terminology used in lab reports	
18. Knowledge of ethical, legal, and social issues related to genetic testing and information recording	
19. Knowledge of Direct-To-Consumer genetic and genomic tests, possible results and potential risks	
Attitudes	
1. Awareness of the sensitivity of genetic information, and the need for privacy and confidentiality while delivering genetic education and counselling	
2. Awareness of the importance of working in a multi-professional team (including the family physician) in evaluation, diagnosis, and treatment of patients tested and referred to genetic consultation	
3. Awareness of the ethical, social, cultural, religious, and ethnic issues that may interfere with care; awareness of the importance of an accurate communication, without coercion or personal bias, and appropriate to the culture, knowledge, and language level of the patient	
Abilities	
1. Ability to gather genetic family history information (including an appropriate multi-generational family history)	
2. Ability to apply the most recent national and international guidelines to manage patients with genetic conditions	
3. Ability to utilize effectively informatic technologies to perform counselling	
4. Ability to understand genetic test results and their clinical implications	
5. Ability to refer the patient to the appropriate experts in genetics and to work in team	
6. Ability to communicate with patients regarding their genetic condition and its implications	
7. Ability to explain basic concepts about probability, disease susceptibility, and the influence of genetic factors on maintenance of health and development of disease	
8. Ability to educate patients about the range of emotions they and/or their family members may experience as a result of receiving genetic information; being able to refer patients to appropriate support groups	
9. Ability to safeguard the privacy and confidentiality of the genetic information of patients	
10. Ability to inform patients of potential limitations of maintaining privacy and confidentiality of genetic information, with an appropriate informed consent process	

As to the curriculum for non-physicians, after the second round, the 33 items proposed were all considered applicable to non-physicians working in genetic services, while just 14 were considered applicable to those not working in genetic services. The 3 new items proposed by the participants were all included into the curriculum for non-physicians working in genetic services after the second round. After the third round, only one new item was modified and included in the curriculum for those not working in genetic services.

In conclusion, we had 15 items (10 of which on knowledge, 3 on attitudes and 2 on abilities) included in the curriculum for non-physician health professionals not working in genetic services, and 36 items included for non-physicians working in genetic services (21 knowledge, 3 attitudes and 12 abilities). Tables 3 and 4 report the complete curriculum for each of the two categories, respectively.

**Table 3:** Core curriculum for non-physicians, identified through the Delphi process

Knowledge	
1. Knowledge of the structure and function of nuclear DNA, genes and chromosomes, their organization into the genome, their replication and transmission through mitosis and meiosis	
2. Knowledge of the structure and regulation of protein-coding genes, their transcription and translation, RNA construction, protein synthesis	
3. Knowledge of the process of DNA mutations (de novo, hereditary); knowledge of the role of these mutations as physiological or pathological events (cancer, multifactorial diseases, monogenic diseases)	
4. Understanding the difference between clinical diagnosis of disease and genetic predisposition to disease. Knowledge of the different types of genetic tests (diagnostic, predictive, test for carriers)	
5. Knowledge of transmission of hereditary diseases (autosomal dominant/recessive, X-linked, mitochondrial, chromosomal, multifactorial)	
6. Knowledge of the organization of genetic services	
7. Knowledge of the potential physical and/or psychosocial benefits and risks of genetic information for individuals in the context of the family and community, here included also the possibility of preventive measures such as reproductive options for mutation carriers	
8. Knowledge of the indications and resources for genetic testing and referral to genetic specialists	
9. Knowledge of ethical, legal, and social issues related to genetic testing and information recording	
10. Knowledge of Direct-To-Consumer genetic and genomic tests, possible results and potential risks	
Attitudes	
1. Awareness of the sensitivity of genetic information, and the need for privacy and confidentiality while delivering genetic education and counselling	
2. Awareness of the importance of working in a multi-professional team (including the family physician) in evaluation, diagnosis, and treatment of patients tested and referred to genetic consultation	
3. Awareness of the ethical, social, cultural, religious, and ethnic issues that may interfere with care; awareness of the importance of an accurate communication, without coercion or personal bias, and appropriate to the culture, knowledge, and language level of the patient	
Abilities	
1. Ability to utilize effectively informatic technologies to perform counselling	
2. Ability to safeguard the privacy and confidentiality of the genetic information of patients

**Table 4:** Core curriculum for non-physicians working in genetic services, identified through the Delphi process

Knowledge	
1. Knowledge of the structure and function of nuclear DNA, genes and chromosomes, their organization into the genome, their replication and transmission through mitosis and meiosis	
2. Knowledge of the structure and regulation of protein-coding genes, their transcription and translation, RNA construction, protein synthesis	
3. Knowledge of the structure, function and transmission of mitochondrial DNA	
4. Knowledge of the process of DNA mutations (de novo, hereditary); knowledge of the role of these mutations as physiological or pathological events (cancer, multifactorial diseases, monogenic diseases)	
5. Understanding the difference between clinical diagnosis of disease and genetic predisposition to disease. Knowledge of the different types of genetic tests (diagnostic, predictive, test for carriers)	
6. Knowledge of transmission of hereditary diseases (autosomal dominant/recessive, X-linked, mitochondrial, chromosomal, multifactorial)	
7. Knowledge of genotype-phenotype correlations; understanding of how gene variations can influence disease presentation, its severity, and clinical manifestation (anticipation, incomplete penetrance, variable expressivity)	
8. Knowledge of the most frequent genetic variants in your professional specialty; knowledge of the clinical features and therapeutic response associated with the different variants	
9. Basic knowledge of the research approaches used to study genomic variants and their correlation with clinical data	
10. Understanding of the importance of the three-generation family history in assessing predisposition to disease	
11. Knowledge of the role of genetic factors in disease prevention	
12. Understanding of the role of behavioural, social, and environmental factors that modify or influence genetics in the manifestation of complex diseases	
13. Knowledge of the organization of genetic services	
14. Knowledge of the potential physical and/or psychosocial benefits and risks of genetic information for individuals in the context of the family and community, here included also the possibility of preventive measures such as reproductive options for mutation carriers	
15. Knowledge of the genetic approaches to treatment (including pharmacogenomics and gene therapy)	
16. Knowledge of the indications for genetic testing and referral to genetic specialists	
17. Knowledge of the principal methodologies for genetic sampling, laboratory techniques with their pros and cons, and knowledge of the terminology used in lab reports	
18. Knowledge of ethical, legal, and social issues related to genetic testing and information recording	
19. Knowledge of Direct-To-Consumer genetic and genomic tests, possible results and potential risks	
20. Function of regulatory factors and epigenetic mechanisms in the regulation of protein-coding genes; role of genetic expression’s regulation in physiological functions and diseases	
21. Methodologies for evaluation of genetic/genomic applications, in terms of effectiveness (analytical validity, clinical validity, clinical utility), cost-effectiveness, and Health Technology Assessment	
Attitudes	
1. Awareness of the sensitivity of genetic information, and the need for privacy and confidentiality while delivering genetic education and counselling	
2. Awareness of the importance of working in a multi-professional team (including the family physician) in evaluation, diagnosis, and treatment of patients tested and referred to genetic consultation	
3. Awareness of the ethical, social, cultural, religious, and ethnic issues that may interfere with care; awareness of the importance of an accurate communication, without coercion or personal bias, and appropriate to the culture, knowledge, and language level of the patient	
Abilities	
1. Ability to gather genetic family history information (including an appropriate multi-generational family history)	
2. Ability to apply the most recent national and international guidelines to manage patients with genetic conditions	
3. Ability to utilize effectively informatic technologies to perform counselling	
4. Ability to understand genetic test results and their clinical implications	
5. Ability to refer the patient to the appropriate experts in genetics and to work in team	
6. Ability to communicate with patients regarding their genetic condition and its implications	
7. Ability to explain basic concepts about probability, disease susceptibility, and the influence of genetic factors on maintenance of health and development of disease	
8. Ability to educate patients about the range of emotions they and/or their family members may experience as a result of receiving genetic information; being able to refer patients to appropriate support groups	
9. Ability to safeguard the privacy and confidentiality of the genetic information of patients	
10. Ability to inform patients of potential limitations of maintaining privacy and confidentiality of genetic information, with an appropriate informed consent process	
11. Ability to perform a reproductive counselling	
12. Ability to transfer genetic competencies to other health professionals and/or facilitate their education in this field	

## Discussion

The aim of our study was to elaborate three curricula containing essential competencies in genetics and genomics, targeted at health professionals not specialized in genetics, a key group still unaddressed as this area of knowledge and practice has been rapidly evolving. To achieve this goal, we performed a literature review in order to identify relevant competencies. Once identified, their inclusion in the three curricula was assessed through a Delphi survey involving Italian experts in the field of genetics and genomics, as participants. We used this procedure for the selection of the items, thanks to the advantages it offers: the anonymity of the Delphi survey prevents the participants from influencing each other; the controlled feedback on the group’s opinion allows the participants to modify the items and propose new ones during the subsequent rounds [21, 22, 27].

We compared the strengths and limitations of our method for identifying core competencies to those published in the literature [14, 19, 28, 29].

The approach chosen to develop these curricula was strengthened through the involvement of participants with a wide range of specialties: geneticists, biologists and physicians as most represented categories. The multidisciplinary composition of the panel was selected with the aim of limiting the risk of a “curriculum overload”, that means avoiding that the contents of the curriculum may be too many or too much specialized [28].

The structure of the curricula (divided into three domains of knowledge, attitudes and abilities) is consistent with other similar studies found in the international literature [14, 19, 29].

The Delphi process was chosen as the best methodology to assess the content of the curricula, similarly to analogous studies with the same aim [19, 29]. The response rate in the first round of our procedure (62.2%) was comparable with other studies that used similar invitation methods (e-mail, post) [19].

While similar studies used a “classical” Delphi methodology [19, 29], we adopted a “modified” Delphi methodology, since the contents of the survey were identified through a literature review. Another considerable difference consists in the target of the curricula: while many studies focused on a specific professional group (mainly on general practitioners) [19, 29], we addressed all healthcare professionals, differentiating the competencies for physicians and for the other healthcare professionals, with a specific distinction for those who work in genetic services. Thanks to this approach, these curricula can be easily incorporated in the post-graduate educational programmes of each professional category. The content of the “knowledge” domain may be taught as frontal or distance teaching lessons; the “abilities” might be transferred through problem-based clinical cases to be solved; the “attitudes” may be part of the ethics teaching contents.

It is remarkable that the three curricula widely differ in the “knowledge” and the “abilities”, while the “attitudes” are the same for every healthcare professional. In particular, physicians and non-physicians who work in genetic services are requested to have the same competencies, while the knowledge and abilities required for non-physicians not working in genetics services should be less detailed. On the other hand, items referring to attitudes were rated as “important” for all the healthcare professionals by the vast majority of participants in the first round, thus suggesting that the relational competencies are considered essential, without distinction as to the professional category.

The main limitation of our study concerns the generalizability of the findings, as we adopted an Italian health system perspective. This led to strict inclusion criteria for the review, excluding papers specifically referred to professional categories not operating in Italy, like non-physician genetic counsellors. These criteria were selected because the validation of the curricula had to be performed by members of the Italian Network for Public Health Genomics. Despite this limitation, we envisage that the outcome of our work, namely the curricula, may be adapted to any professional categories. A limitation may also be found in reaching a consensus during the Delphi process: while in the first round the response rate was 62.2%, in the third round it decreased to 32.4%. Another limitation concerns the selection of the survey participants: even if a multi-professional and multidisciplinary group was involved, only experts in genetics participated in the Delphi process. Lack of different perspectives could be addressed in the future by submitting the curricula to a wider target group for further validation and by involving non-experts in genetics and patient representatives.

## Conclusions

We identified the contents of three curricula in genetics for non-genetic health professionals, differentiating those who work and who do not work in genetic services. These curricula are intended as an exhaustive and ready-to-use material for post-graduate courses about genetics/genomics. The implementation of these competencies in an educational programme is made feasible thanks to the structure of the curricula divided into the three mentioned domains.

The relevance of our results is related to the urgent need for improving the genetics/genomics literacy of healthcare professionals who are not specialized in genetics, as a possible response to the mainstream spread of this kind of knowledge and practice [30].

## Abbreviations

ESHG: European Society of Human Genetics
GENISAP: Italian Network for Public Health Genomics
NCHPEG: National Coalition for Health Professional Education in Genetics
UK NHS NGGEC: UK National Health Service National Genetics and Genomics Education Centre
